# Self-Reported Psychosis Spectrum Symptoms Among Sexual and Gender Diverse Emerging Adults Screened for a Suicide Prevention Trial

**DOI:** 10.1007/s10508-026-03438-w

**Published:** 2026-04-24

**Authors:** Jennifer Ben Nathan, Lindiwe Mayinja, Jessica L. Webster, Jennifer T. Tran, Tyler Burgese, Monica E. Calkins, Maria A. Oquendo, Gregory Brown, David Mandell, José A. Bauermeister, Lily A. Brown

**Affiliations:** 1 Department of Family and Community Health, School of Nursing, University of Pennsylvania, Philadelphia, PA USA; 2 Department of Psychiatry, Perelman School of Medicine, University of Pennsylvania, 3535 Market Street, Suite 600 N, Philadelphia, PA 19104 USA

**Keywords:** Psychosis spectrum, Identity, Young adults, Gender identity, Sexual orientation

## Abstract

Psychosis spectrum symptoms, including a range of unusual thoughts and perceptual abnormalities, are markers of increased vulnerability to psychotic disorders. Sexual and gender diverse (SGD) populations experience higher rates of psychotic disorders than the general population, raising the possibility that emerging adult SGD individuals may also report elevated psychosis spectrum experiences. We hypothesized that SGD emerging adults would report higher rates of psychosis spectrum symptoms than the general population. In line with intersectional frameworks, we further hypothesized that rates would be higher among individuals with multiple marginalized identities. We screened SGD emerging adults (ages 18–24; *N* = 376) for a clinical trial of a mobile health intervention to decrease suicide risk. A brief eligibility screening questionnaire assessed racial, ethnic, sexual, and gender identities. The Diagnostic Interview for Anxiety, Mood, and OCD and Related Neuropsychiatric Disorders (DIAMOND) psychosis screener questions were used to assess self-reported unusual thoughts and perceptual abnormalities. Psychosis spectrum symptoms were reported by 40% of the screened sample versus 23% in the general population. The probability of endorsing symptoms (OR = 1.528, 95% CI = 1.16–1.99, *p* = 0.002) increased with each additional minority status identification (i.e., race, ethnicity, sexual identity, gender identity). Future research should explore unique factors contributing to the heightened rates of self-reported psychosis spectrum symptoms among these minority populations and attempt to test the replicability of these findings.

**Supplementary Information:**

The online version contains supplementary material available at 10.1007/s10508-026-03438-w.

## Introduction

Psychosis spectrum symptoms include unusual thoughts and perceptual abnormalities that occur across a continuum of clinical significance and symptom severity ranging from psychotic experiences, clinical high-risk syndromes, and schizophrenia spectrum and mood disorders (Jeste & Green, 2025). A recent systematic review and meta-analysis found that approximately 2% of individuals report new onset of psychotic experiences annually (Staines et al., 2023). Although most individuals with psychotic experiences do not go on to develop a psychotic disorder (Linscott & van Os, 2010), having psychotic experiences is associated with a fourfold increased risk of developing a psychotic disorder and a threefold increased risk of developing any non-psychotic mental disorder (Healy et al., 2019). Psychotic experiences are also associated with numerous adverse outcomes including, but not limited to, chronic physical health conditions (Oh et al., 2021; Scott et al., 2018), disability (Navarro‐Mateu et al., 2017), loneliness (Narita et al., 2022), and suicidal ideation and behaviors (Yates et al., 2019). Risk for psychotic illness is also elevated among racial and ethnic minority individuals, potentially due to significant biases in assessment, diagnosis, and healthcare management (van der Ven et al., 2024). Thus, biological and social factors undoubtably increase risk for psychosis (Murray et al., 2020) though the exact combination and dosage of risk factors required to instantiate psychosis spectrum symptoms remain unclear.

Sexual and gender diverse (SGD) populations are at greater risk for psychotic disorders than their cisgender, heterosexual peers (Barr et al., 2021; Bolton & Sareen, 2011; Chakraborty et al., 2011; Hanna et al., 2019). In the general population, identifying as a sexual minority was associated with between 1.9–2.6 times higher odds for psychotic experiences (Gevonden et al., 2014; Jacob et al., 2021; Oh et al., 2024). In an online sample, sexual and gender minority participants had higher scores of schizophrenia-like experiences than non-sexual and gender minority participants (Hammer et al., 2025). Recent studies in adolescents reported that gender diverse youth and sexual diverse youth had 2.3 and 2.5 times higher odds, respectively, of psychotic-like experiences versus hetero/cisgender youth (Pachas et al., 2025). The higher prevalence of poor mental health outcomes among SGD people can be attributed to the social stressors (i.e., stigma and discrimination), as well as significant biases in assessment, diagnosis, and healthcare management experienced by this population (Meyer, 2003).

Few studies have focused on psychotic experiences among sexual and gender minority emerging adults (18–24 years old), when the risk of developing a psychotic disorder is particularly high (Solmi et al., 2022). While estimates on prevalence rates of psychotic experiences vary in younger population samples, a meta-analysis on childhood and adolescent psychotic experiences found that 9.8% of participants reported psychosis symptoms (Healy et al., 2019), and a meta-analysis of teen and young adult students revealed that 23.31% of students reported having experienced psychosis symptoms (Fekih–Romdhane et al., 2022). Greater endorsement of psychotic experiences during emerging adulthood is predictive of subsequent psychotic disorders (*hazard ratio* (*HR*) = 1.76, 95% CI = 1.11–2.80, *p* = .016) and any mental disorder including psychotic disorders (*HR* = 1.39, 95% CI = 1.16–1.67, *p* < .001; Lindgren et al., 2022), indicating that emerging adulthood is a critical time to study.

Furthermore, there is a paucity of research examining how intersecting marginalized identities—defined as the co-occurrence of multiple historically oppressed social statuses—shape risk for psychosis spectrum symptoms within SGD populations. Intersectionality theory posits that systems of oppression do not operate independently or additively but rather interact to produce qualitatively distinct forms of marginalization and stress exposure (Rivas–Koehl et al., 2023). Empirical work supports this framework: individuals holding both sexual and gender minority identities demonstrate poorer mental health outcomes than those with a single minority identity (Borgogna et al., 2019), and Black sexual minority women report worse mental health than their White sexual minority counterparts (Calabrese et al., 2015). These findings suggest that risk cannot be fully understood by examining single-axis identities in isolation. This gap is particularly consequential for emerging adulthood, a developmental period characterized by heightened stress sensitivity and ongoing identity consolidation (Arnett, 2000). The convergence of multiple minoritized statuses during this stage may intensify exposure to chronic and acute psychosocial stressors (e.g., discrimination, vigilance, exclusion), which are well-established contributors to psychotic experiences (Brandt et al., 2022). Thus, an intersectional approach is critical for identifying subgroups of SGD emerging adults who may be at especially elevated risk.

The purpose of the current study was to build on existing research establishing higher prevalence of psychotic experiences among emerging adult SGD individuals (e.g., Corcoran et al., 2024; Wittgens et al., 2022) by evaluating the prevalence of self-reported psychosis spectrum symptoms among an emerging adult sample that includes individuals with multiple marginalized identities. Further examination of the link between psychosis spectrum symptoms and having a minoritized sexual and/or gender identity is warranted given the need to elucidate factors that might explain this phenomenon and given evidence that individuals who are members of multiple minoritized groups may be at greater risk (Barr et al., 2021; Termorshuizen et al., 2023). In line with prior literature demonstrating elevated risk for psychotic experiences among SGD individuals, we hypothesized that rates of self-reported psychosis spectrum symptoms would be greater than published prevalence rates in the general population of emerging adults (e.g., 23.31% of teen and young adult students; Fekih–Romdhane et al., 2022). We also aimed to examine the association between multiple minority status and rates of psychosis spectrum symptoms. Given that multiple intersecting minoritized identities are a risk factor for deleterious health outcomes per intersectionality theory (Rivas–Koehl et al., 2023), we hypothesized that rates of self-reported psychosis spectrum symptoms would be elevated among individuals with multiple minoritized identities.

## Method

### Participants

This study used data from individuals screened to participate in a randomized control trial (NCT05018143) evaluating the efficacy of a digital health intervention to reduce suicidal ideation and behaviors among SGD emerging adults (ages 18–24). Detailed information on the clinical trial protocol has been published elsewhere (Brown et al., 2023). Our study only included participants (*N* = 376) between the ages of 18–24 who identified as a sexual and/or gender minority.

### Procedures

Participants were recruited through several means: a social media campaign, physical flyers posted in the local community, and a virtual flyer distribution to email listservs. To determine eligibility for the clinical trial, interested individuals clicked a link to an online self-report screener via a social media recruitment campaign or QR code on a physical flyer. A majority of our screeners were from an Instagram ad (64.2%), followed by Facebook ads (14.6%), and from study flyers (10.4%). The social media campaign was created on Facebook and Instagram from July 2023 to October 2023. Ads targeted users within the 18–24 year age range and located within a 10-mile radius around Philadelphia County (i.e., people living in or recently in this location based on a combination of device location information, IP address, and GPS coordinates).

Ad delivery was optimized using Meta Ad Manager’s ‘Traffic’ objective, which prioritizes showing ads to users predicted by the platform’s algorithm to be most likely to click the link. This prediction is based on user engagement history, demographics, and behavioral signals. Ads were targeted broadly by age and location, but the algorithm dynamically adjusted delivery to maximize link clicks.

Community listservs serving SGD emerging adults were contacted to send out a virtual flyer to their community members. Physical flyers were posted across Philadelphia County with permission of businesses and institutions. The social media campaign and flyers included language required from the IRB and a conversational tone communicating important aspects of the study, including compensation, study focus, and eligibility. To prevent forms from being filled out by fraudulent bots, Google reCAPTCHA (a Turing test to distinguish between humans and bots) was enabled within REDCap to protect the public survey link from fraudulent survey completion by automated software.

The screener survey included initial consent to be screened followed by preliminary eligibility questions. Participants were asked their age, gender identity, sexual identity, if they lived in the Philadelphia metropolitan area and planned to live in the area for the next 6 months, and if they possessed a smartphone. Race, ethnicity, and primary language were also assessed but not utilized to determine eligibility for the larger study. A copy of the screener is included as supplemental material. Data were collected and managed using REDCap (Harris et al., 2009, 2019).

### Measures

#### Self-Reported Psychosis Spectrum Symptoms

**Fig. 1:**
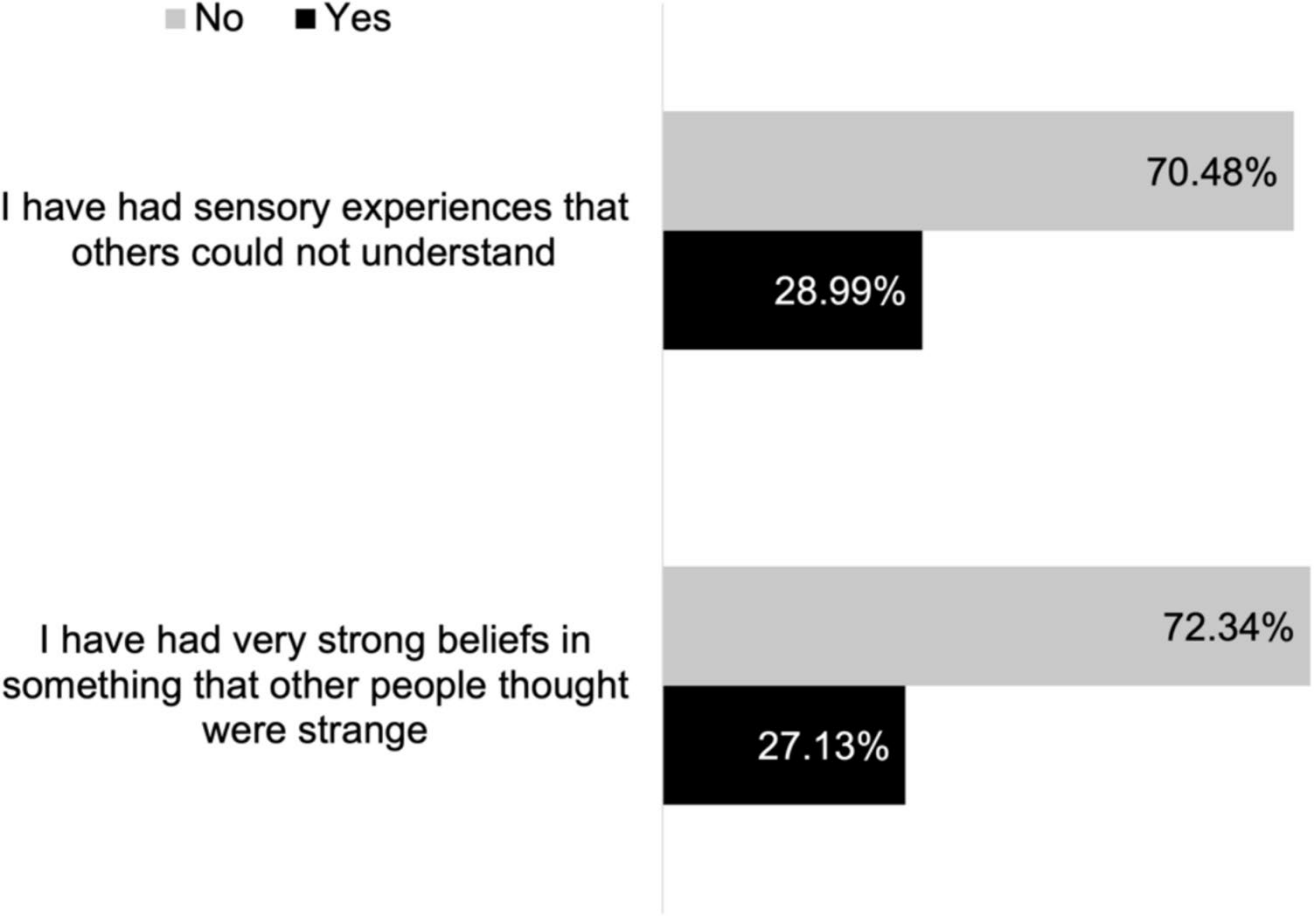
Graph of frequency distributions of yes/no to Perceptual Abnormalities and Unusual Thoughts

**Fig. 2:**
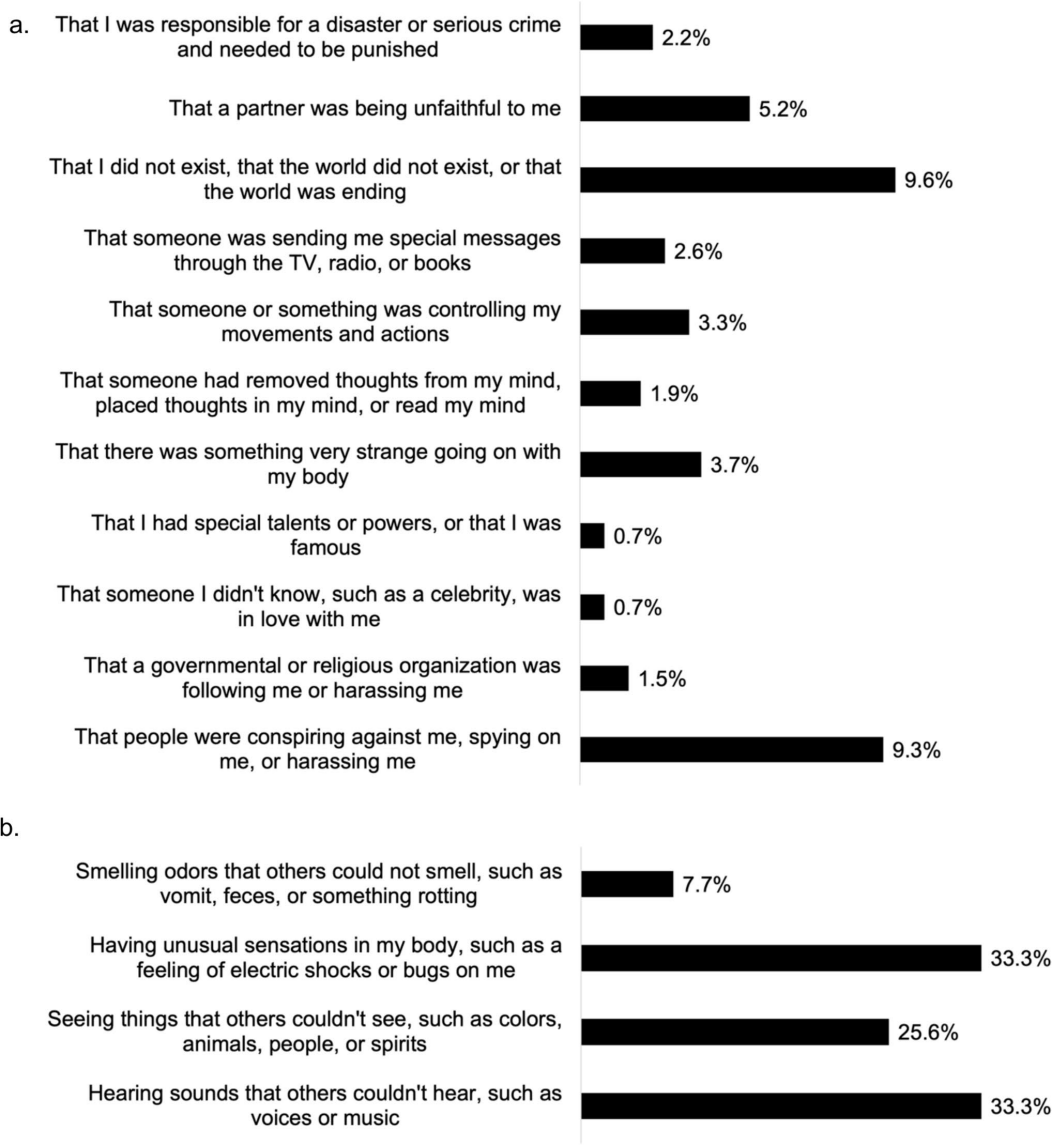
Percentages of endorsed example items in unusual thoughts **a** and perceptual abnormalities **b**

Participants were screened for self-reported psychosis using two questions obtained from the Diagnostic Interview for Anxiety, Mood, and OCD and Related Neuropsychiatric Disorders (DIAMOND) screener. The DIAMOND interview, which includes both the screener and modules assessing individual disorders, has demonstrated good reliability and validity in adult clinical samples (Tolin et al., 2018). For all diagnoses, interrater reliability ranged from very good to excellent (κ = 0.62–1.00), test–retest reliability ranged from good to excellent (κ = 0.59–1.00), and convergent validity ranged from moderate to very high (Cohen’s *d* = 0.52–4.80; Tolin et al., 2018). The two DIAMOND questions (Figs. 1 and 2) screen for self-reported potentially unusual thoughts (hereafter referred to as unusual thoughts, e.g., “That someone had removed thoughts from my mind, placed thoughts in my mind, or read my mind”, “That someone was sending me special messages through the TV, radio, or books”) and perceptual abnormalities (e.g., “Hearing sounds that others couldn't hear”, “Seeing things that others couldn’t see”) using binary (yes/no) answer categories.

The psychosis screener underwent two sequential modifications to obtain more detailed information from participants who endorsed psychotic experiences. Initially, all participants (*N* = 376) completed the original screener, which required dichotomous (“yes”/ “no”) responses to two items assessing perceptual abnormalities and unusual thoughts. The first modification added a free-text response option following endorsement of unusual thoughts, allowing participants to describe their experiences in their own words (completed by a subset of 270 participants); this free-text option was later extended to the perceptual abnormalities item (completed by a subset of 39 participants). The second modification added a “check all that apply” checklist following endorsement of either perceptual abnormalities or unusual thoughts to capture specific symptom content areas (completed by the same subset of 39 participants).

Free-text responses describing unusual thoughts were independently reviewed by two members of the research team, who categorized each response into one or more of the 11 example categories provided by the DIAMOND assessment. Categorizations were compared to assess consistency, and all discrepancies were resolved through discussion, resulting in 100% agreement.

To evaluate whether the screener modifications introduced systematic differences across participants, a series of one-way ANOVA and chi-square analyses compared demographic and symptom endorsement characteristics across the three screener versions. There were no significant differences in age (*p* = 0.129), race (*p* = 0.126), or ethnicity (*p* = 0.214). Additionally, rates of self-reported unusual thoughts (*p* = 0.139), perceptual abnormalities (*p* = 0.812), and endorsement of any psychosis spectrum symptoms (*p* = 0.933) did not differ across screener versions.

#### Sociodemographic Characteristics and Cumulative Minoritized Identity Score

Participants completed an assessment of demographics, including racial identity, Hispanic ethnicity, sexual identity, and gender identity (see Table 1). Using these questions, we computed a cumulative social identity disadvantage score (Teshome et al., 2022). Each participant was assigned a value (1–4) to reflect the possession of one or more minoritized identities: racial, ethnic, sexual, and gender. Participants who self-reported belonging to any race other than White received a point in the racial minority category. Participants who reported being Hispanic, Latino, Latinx, or of Spanish heritage received a point in the ethnic minority category regardless of racial identification (Hitlin et al., 2007). We scored the minoritized data (racial minority = 0/1, ethnic minority = 0/1) to align with the U.S. census, though Hispanic people increasingly advocate for their ethnicity to be a racial category (Noé-Bustamante et al., 2021). Participants who reported any sexual identity other than heterosexual received a point for the sexual minority category. Participants who self-reported their gender identity as agender, genderqueer, non-binary, transgender man, transgender woman, questioning, and/or another gender received a point for gender minority.

**Table 1:** Sociodemographic characteristics of the sample and rates of endorsement of self-reported psychosis screening questions

	All	Endorsed self-reported psychosis screening questions				
	*n* (%)	n (% of total sample)	% of row	UT only	PerAb only	Both
Total sample	376 (100.0%)	151 (40.2%)	40.2%	42	49	60
*Race*						
American Indian or Alaska native	3 (0.8%)	1 (0.7%)	33.3%	0	0	1
Asian	62 (16.5%)	16 (10.6%)	25.8%	7	4	5
Black or African American	54 (14.4%)	29 (19.2%)	53.7%	6	9	14
Native Hawaiian or other Pacific Islander	1 (0.3%)	1 (0.7%)	100.0%	0	0	1
White	214 (56.9%)	82 (54.3%)	38.3%	22	29	31
Other	15 (4.0%)	10 (6.6%)	66.7%	3	2	5
More than one race	27 (7.2%)	12 (7.9%)	44.4%	4	5	3
***Ethnicity***						
Hispanic, Latino, Latinx, or of Spanish heritage	49 (13.0%)	24 (15.9%)	49.0%	9	6	9
***Gender identity***						
Agender	9 (2.4%)	3 (2.0%)	33.3%	0	1	2
Cisgender man	52 (13.8%)	14 (9.3%)	26.9%	4	5	5
Cisgender woman	77 (20.5%)	25 (16.6%)	32.5%	10	5	10
Genderqueer	38 (10.1%)	17 (11.3%)	44.7%	5	5	7
Man	48 (12.8%)	14 (9.3%)	29.2%	2	5	7
Woman	86 (22.9%)	35 (23.2%)	40.7%	9	13	13
Non-binary	89 (23.7%)	47 (31.1%)	52.8%	12	14	21
Transgender man	58 (15.4%)	28 (18.5%)	48.3%	6	9	13
Transgender woman	19 (5.1%)	10 (6.6%)	52.6%	3	3	4
Questioning	21 (5.6%)	10 (6.6%)	47.6%	1	5	4
Another	8 (2.1%)	1 (0.7%)	12.5%	0	1	0
***Sexual identity***						
Bisexual	149 (39.6%)	62 (41.1%)	41.6%	19	19	24
Gay	85 (22.6%)	28 (18.5%)	32.9%	10	5	13
Lesbian	59 (15.7%)	22 (14.6%)	37.3%	2	8	12
Straight, that is, not gay or lesbian, etc	2 (0.5%)	1 (0.7%)	50.0%	1	0	0
None of these describe me, and I'd like to see additional options	81 (21.5%)	38 (25.2%)	46.9%	10	17	11

UT, unusual thoughts; PerAB, perceptual abnormalities

### Data Analysis

Our first objective was to examine the rates of psychosis spectrum symptoms in our sample. We calculated this utilizing a percentage of participants who endorsed psychosis spectrum symptoms (based on the two questions asking about experiences of unusual thoughts and perceptual abnormalities) out of the total sample (*N* = 376). The prevalence of psychosis spectrum symptoms in our sample was similarly calculated for the exploratory analyses. In order to test our hypothesis that multiple minority identities were associated with psychosis symptoms, we conducted a logistic regression analysis in Stata 17 (StataCorp, College Station, TX) to examine the association between possession of minority identities (independent variable) and self-reported psychosis spectrum symptoms (dependent variable), following a cumulative marginalized identities count variable used in prior research (Teshome et al., 2022; Wang et al., 2024).

## Results

### Self-Reported Psychosis Spectrum Symptoms

Of the total sample (*N* = 376), 102 (27.13%) endorsed experiencing unusual thoughts and 109 (28.99%) endorsed experiencing perceptual abnormalities (Fig. 1). In total, 40.16% of the sample self-reported some psychosis spectrum symptom, endorsing one or both DIAMOND screening items. Among the subset of participants asked to provide free text responses elaborating on endorsed experiences (*n* = 270), types of unusual thought content endorsed were most commonly categorized as beliefs that: (1) people were conspiring against me, spying on me, or harassing me; and (2) I did not exist, that the world did not exist, or that the world was ending. Figure 2 displays percentages of particular beliefs and sensory experiences endorsed by the subset of participants administered the specifier questions (*n* = 270 and *n* = 39, respectively). Consistent with the results of free text categorizations, the most commonly reported beliefs with the “check all that apply” questions included that: (1) people were conspiring against me, spying on me, or harassing me; and (2) I did not exist, that the world did not exist, or that the world was ending. Auditory and somatic/tactile perceptual experiences were the most frequently endorsed sensory experiences.

### Multiple Minoritized Identity Status and Self-Reported Psychosis

The sample consisted of 162 (43.09%) participants who identified as a racial minority, 49 (13.03%) ethnic minority, 177 (47.07%) gender minority, and 374 (99.47%) sexual minority identity. A chi-square analysis demonstrated that gender minority status was associated with reporting psychosis symptoms (*p* < 0.01), where participants who identified as a gender minority were more likely to report psychosis symptoms (48.6%) than those who did not (32.7%). In contrast, racial and ethnic minority status were not associated with reporting psychosis symptoms (*p* = 0.402 and 0.177, respectively).

**Fig. 3:**
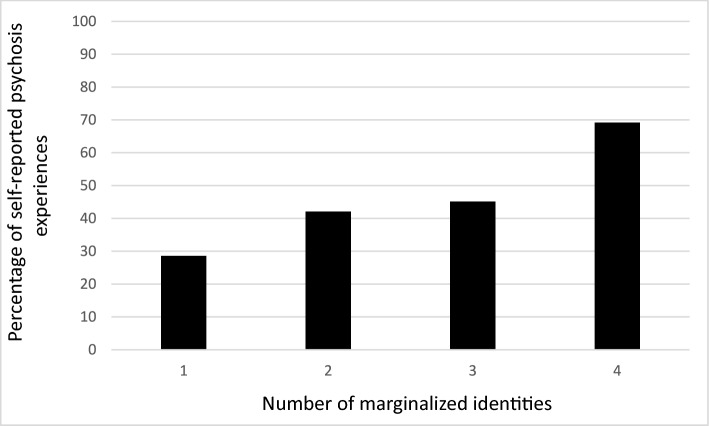
Multiple marginalized identity status and self-reported psychosis experiences (%)

Among participants reporting one minoritized identity (*n* = 98), 28 (28.57%) reported psychosis symptoms, with rates increasing for each additional minority status (Fig. 3). A logistic regression analysis revealed that holding multiple minoritized identities was associated with increased odds of reporting psychosis symptoms (*OR* = 1.52, 95% CI = 1.16–1.99, *p* = 0.002).

## Discussion

Emerging adulthood is characterized by numerous biological, psychological, and social changes that have been implicated in the onset of psychosis symptoms, including ongoing brain maturation (O’Hanlon et al., 2015; Patel et al., 2021), hormonal changes (Trotman et al., 2013), and heightened exposure to social stressors (Liu et al., 2021). Although the present study did not include a direct comparison group, the observed prevalence of self-reported psychosis spectrum symptoms among SGD participants (40%) appears substantially higher than estimates from general population samples (23%). Testing replicability of findings in studies with appropriate comparison groups is necessary to determine whether this elevation is robust and to clarify the mechanisms contributing to heightened risk for psychosis spectrum symptoms among SGD emerging adults.

Consistent with our hypotheses and informed by the intersectionality theory (Rivas‐Koehl et al., 2023), the likelihood of endorsing psychosis spectrum symptoms increased with each additional minoritized identity (i.e., race, ethnicity, sexual orientation, and gender). Importantly, this pattern may reflect the influence of multiple, overlapping social determinants of health such as access to mental health care, socioeconomic conditions, and exposure to environmental stressors, which are correlated with, but not reducible to, minoritized identity categories. Accordingly, these findings should not be interpreted as evidence of a singular explanatory pathway. Rather, they highlight the need for intersectional approaches that can account for how multiple structural and social factors may jointly shape vulnerability during a sensitive developmental period (Presseau et al., 2022).

Minority stress processes represent one plausible contributor to the elevated rates of psychosis symptoms observed among SGD individuals. Prior research indicates that SGD populations experience higher levels of discrimination, victimization, and social adversity than the general population (Bond et al., 2021; Chakraborty et al., 2011), and that such experiences are associated with increased risk for severe mental illness, including non-affective psychotic disorders (Kidd et al., 2016; Post et al., 2021; Schneeberger et al., 2014). In this study, elevated rates of persecutory beliefs may be consistent with the possibility that discrimination-related experiences contribute to some psychosis spectrum symptoms. However, the endorsement of symptoms less directly tied to social threat, such as nihilistic beliefs and auditory or somatic hallucinations, suggests that minority stress alone is unlikely to fully account for the observed symptom profile.

Other pathways, including early-life adversity and trauma exposure, may also play a critical role. Childhood trauma and abuse are commonly reported among individuals with psychosis symptoms and are associated with substantially increased risk of psychosis in adulthood (Bendall et al., 2007; Varese et al., 2012). Given evidence that SGD individuals report elevated exposure to such adversities (Blosnich & Andersen, 2015; Brew et al., 2018), future prospective research is needed to disentangle how developmental, biological, trauma-related, and minority stress processes may interact over time to shape psychosis risk. Longitudinal studies incorporating direct measures of discrimination, trauma, and structural determinants of health will be essential for clarifying these mechanisms.

Evidence for increased risk of psychosis symptoms among the SGD population has vital clinical implications for clinicians who treat this population. In the present study, 27% of participants reported unusual thought content and 29% reported perceptual abnormalities. Regularly screening SGD clients for psychosis spectrum symptoms may be a useful practice, as endorsement of these symptoms can be an indicator that further assessment and exploration of the clients’ experiences is warranted to better target their potential mental and physical health treatment needs.

### Limitations and Future Directions

This study has several limitations that warrant consideration. First, participants were recruited based on self-reported suicidal ideation, which likely increased the prevalence of symptom endorsement, given the well-established association between psychosis and suicide risk (Bornheimer et al., 2024). In addition, a convenience sample recruited via social media may limit generalizability. Because advertisement delivery was optimized for link clicks, participants may differ systematically from the broader population of social media users, with potential overrepresentation of individuals who are more likely to engage with online ads or research studies. This self-selection bias may limit the generalizability of prevalence estimates. Furthermore, participants were categorized into racially and ethnically diverse groups for analyses in this study, which might have obscured important differences in identity that should be the focus of future work.

Second, participants who were deemed ineligible for the suicide risk–reduction intervention did not complete a clinical interview eligibility assessment, nor did we ask them about sex at birth on the screener because this question can evoke distress that we wanted to avoid during an online screener (whereas we assessed this for enrolled participants). Consequently, we could not assess clinical features of endorsed experiences such as content, tenacity/conviction, distress, interference, frequency, and duration required to determine clinical significance and severity, nor their context, including potential substance use and general medical conditions. We were therefore also unable to determine whether participants met criteria for a clinical high-risk syndrome or a DSM-5 clinical disorder (e.g., schizophrenia spectrum, mood disorder with psychosis, substance-induced psychosis). These limitations notwithstanding, the observed self-reported symptom rate is meaningful because, based on the extant literature, it increases the risk of poor mental health outcomes in this vulnerable population.

Third, estimates of self-reported psychotic experiences vary across studies as a function of the assessment instrument used (Lee et al., 2016). In this study, the DIAMOND psychosis screener was selected as a brief, structured measure appropriate for clinical trial enrollment and for identifying the presence of psychosis spectrum symptoms at a screening level. The DIAMOND has been widely used in clinical research settings and includes standardized items assessing psychosis spectrum symptoms, making it well suited to the study’s primary aim of estimating the prevalence of endorsed psychosis spectrum symptoms rather than characterizing symptom severity, duration, or functional impairment. The brief self-report screener, however, does not assess the frequency or impact of symptoms, nor does it capture gradations of psychosis spectrum severity. Future research building on these findings could incorporate complementary self-report measures—such as the Prodromal Questionnaire–Brief (PQ-B; Kline & Schiffman, 2014; Kline et al., 2012; Loewy et al., 2011) or the PRIME Screen–Revised (PS-R; Calkins et al., 2025; Kobayashi et al., 2008; Miller et al., 2004)—to provide more fine-grained characterization of psychosis-related phenomena.

Importantly, self-report screeners for psychotic experiences, including the PQ-B and PS-R, do not directly assess contextual and sociocultural factors. For example, items assessing suspiciousness or perceived persecution may reflect real-world experiences of discrimination, harassment, or interpersonal stress, particularly among marginalized populations (Bridgwater et al., 2023). Incorporating follow-up probes or multimethod assessment approaches may therefore enhance interpretability across instruments. Taken together, these considerations suggest that the present findings are best understood as reflecting the presence of psychosis spectrum symptoms at a screening level, while identifying directions for future methodological refinement rather than indicating a fundamental limitation of the study.

Despite these limitations, the study also has notable strengths. The addition of follow-up survey items allowed for greater insight into participants’ endorsements of psychosis spectrum symptoms and was implemented in response to unexpectedly high rates of symptom endorsement observed during recruitment. These additions enabled participants to describe their experiences and to specify the types of symptoms endorsed. Preliminary findings from these added items support the utility of incorporating follow-up questions when screening for psychosis spectrum symptoms, particularly in community-based or high-risk samples.

### Conclusion

The current study found high rates of endorsement of self-reported psychosis spectrum symptoms among participants who completed an online screen for a study focused on suicide prevention among SGD emerging adults. With each increase in identification with a minoritized identity, rates of endorsement of psychosis spectrum symptoms increased. This suggests the possibility that experiences of minority stress might contribute to the increased risk of psychotic experiences among the SGD population. These findings require replication in a larger, more representative sample with a comparison group and confirmation by clinical interview. These findings highlight the need to thoroughly assess psychosis symptoms among SGD individuals.

## Supplementary Information

Below is the link to the electronic supplementary material.

Supplementary file1 (PDF 154 KB)

Supplementary file2 (PNG 6741 KB)

Supplementary file3 (PNG 16491 KB)

## Data Availability

Available upon request.

## Code Availability

Available upon request.
