# Supplementary material for: Self-Reported Psychosis Spectrum Symptoms Among Sexual and Gender Diverse Emerging Adults Screened for a Suicide Prevention Trial
Source: Arch Sex Behav. 2026 Apr 24;55(4):1455–65. doi: 10.1007/s10508-026-03438-w (PMC13275789; doi:10.1007/s10508-026-03438-w)
Supplement: Supplementary file 1 — Supplementary file1 (PDF 154 KB) [file 10508_2026_3438_MOESM1_ESM.pdf]

Interested in the STARS Project?

Please complete the survey below.

Thank you!

Please review this brief consent statement, and indicate your interest in continuing at the end of the form.

(On a mobile device, you may need to scroll to the bottom to see the question.)

CONSENT TO BE SCREENED FOR ELIGIBILITY IN A RESEARCH STUDY

You are invited to take a short survey to see if you qualify for a research study. The study is a test of a suicide prevention program for LGBTQ+ young adults. This study is being conducted at the University of Pennsylvania School of Nursing (PIs José Bauermeister and Lily Brown). Before you can join the study, we will need to make sure you qualify. You will need to take a short survey that asks questions about your demographic characteristics, where you live, and other health-related topics. These questions will take about 5-10 minutes to answer.

If you qualify to be in the study, we will ask for contact information which the study team will use to contact you to provide more information about the study and give you the opportunity to choose whether or not to consent to participate. You will receive another consent form that will explain the details of the rest of the study.

Taking this survey is voluntary. If you don't want to take this survey, you can close this browser screen now. There is no direct benefit to you for completing the survey.

Whether you join the study or not, the information you provide in this survey may be seen by researchers at Penn, any sponsor of the study, and those responsible for oversight of the study. We try to make sure that the information we collect from you is kept private and used only for the research study we are discussing. If you do not agree to continue the eligibility screening survey, it will not affect your current or future care at Penn.

CONTACT INFORMATION
To find out more about the study, to ask a question or express a concern about the study, or to talk about any problems you may have as a study subject, you may contact the STARS study team at the University of Pennsylvania at 215-898-9054 or STARSstudy@nursing.upenn.edu.

If a member of the research team cannot be reached or you want to talk to someone other than those working on the study, you may contact the Office of Regulatory Affairs with any questions, concerns, or complaints at the University of Pennsylvania by calling (215) 898-2614.

Do you agree to take this screening survey to see if you qualify for the study?

- ◯ Yes, I would like to see if I am eligible for this study.
- ◯ I DO NOT consent to participate in the screening survey

Which of the following best represents how you think of yourself?	◯ Gay<br>◯ Lesbian<br>◯ Straight, that is, not gay or lesbian, etc.<br>◯ Bisexual<br>◯ None of these describe me, and I'd like to see additional options
Are any of these a closer description of how you think of yourself?	◯ Queer<br>◯ Polysexual<br>◯ Omnisexual, sapiosexual or pansexual<br>◯ Asexual<br>◯ Two-spirit<br>◯ Pansexual<br>◯ Have not figured out or are in the process of figuring out your sexuality<br>◯ Do not think of yourself as having sexuality<br>◯ Do not use labels to identify yourself<br>◯ Don't know the answer<br>◯ No, I mean something else<br>◯ Prefer not to answer
If you answered "No, I mean something else," please specify:	__________________________________
SOGI Identity Inclusion	__________________________________
Do you have a smartphone?	◯ Yes<br>◯ No
Do you currently live in Pennsylvania metropolitan area?	◯ Yes<br>◯ No
What is the zip code of your current residence?	__________________________________
Do you plan to move out of the Pennsylvania metropolitan area within the next 6 months?	◯ Yes<br>◯ No
When will you be moving out of the Pennsylvania area? (month)	__________________________________

Please read the following statements and select YES or NO to indicate whether each statement applies to you. If you are not sure whether a statement applies to you, select YES.

I have had sensory experiences that others could not understand, such as:<br><br>a. Hearing sounds that others couldn't hear, such as voices or music<br>b. Seeing things that others couldn't see, such as colors, animals, people, or spirits<br>c. Having unusual sensations in my body, such as a feeling of electric shocks or bugs on me<br>d. Smelling odors that others could not smell, such as vomit, feces, or something rotting	◯ Yes<br>◯ No<br><br>If yes, select all that apply.

Please Describe:

____

I have had very strong beliefs in something that other people thought were strange, such as any of the following:

a. That people were conspiring against me, spying on me, or harassing me
b. That a governmental or religious organization was following me or harassing me
c. That someone I didn't know, such as a celebrity, was in love with me
d. That I had special talents or powers, or that I was famous
e. That there was something very strange going on with my body
f. That someone had removed thoughts from my mind, placed thoughts in my mind, or read my mind
g. That someone or something was controlling my movements and actions
h. That someone was sending me special messages through the TV, radio, or books
i. That I did not exist, that the world did not exist, or that the world was ending
j. That a partner was being unfaithful to me
k. That I was responsible for a disaster or serious crime and needed to be punished

◯ Yes
◯ No

If yes, select all that apply.

Please describe:

____

In the past month, have you actually had any thoughts of killing yourself?

◯ Yes
◯ No

Thank you for completing the survey. You may be eligible to participate in the STARS study. Please provide contact information for a brief follow-up in which we will describe the study and schedule an appointment, and someone from our team will reach out within one business day.

What is your first name?

____

What is your last name?

____

What is your email address?

____

What is your phone number?

____

How may we contact you about the study? (Check all that apply)

☐ Phone
☐ Text
☐ Email
☐ I changed my mind and I do not want to be contacted about participating

Who is your phone carrier for text messaging? (Note: text messaging rates may apply) __________________________________

What days and times are you available for a phone call?

	Mornings (8 AM - 12 PM)	Afternoons (12 PM - 5:00 PM)	Evenings (5 PM - 8 PM)	None
Monday	☐	☐	☐	☐
Tuesday	☐	☐	☐	☐
Wednesday	☐	☐	☐	☐
Thursday	☐	☐	☐	☐
Friday	☐	☐	☐	☐

Is it okay for us to leave a voicemail?

◯ Yes
◯ No

How did you hear about this study?

☐ Instagram
☐ Facebook
☐ Dating Website
☐ Study Flyer or Postcard
☐ Medical Doctor
☐ Mental Health Provider
☐ Friend or Family
☐ Other
☐ Prefer not to answer

What dating website did you about this study from?

☐ Her
☐ Lex
☐ WAPA
☐ Other

What was the dating website? __________________________________

If other, tell us where you heard about the study ____

Do we have permission to contact you for future studies?

◯ Yes
◯ No

If you would like to learn more about other study opportunities that you may be eligible for in the future, please click here or copy and paste the link below:

https://upenn.co1.qualtrics.com/jfe/form/SV_8cUBaEc2f1TVnMN

Thank you for completing this screener survey! If you have any questions or comments regarding this survey, please provide them here. ____

Attached is a list of resources.

[Attachment: "Resources.pdf"]
