# Supplementary figures and images for: Self-Reported Psychosis Spectrum Symptoms Among Sexual and Gender Diverse Emerging Adults Screened for a Suicide Prevention Trial
Source: Arch Sex Behav. 2026 Apr 24;55(4):1455–65. doi: 10.1007/s10508-026-03438-w (PMC13275789; doi:10.1007/s10508-026-03438-w)

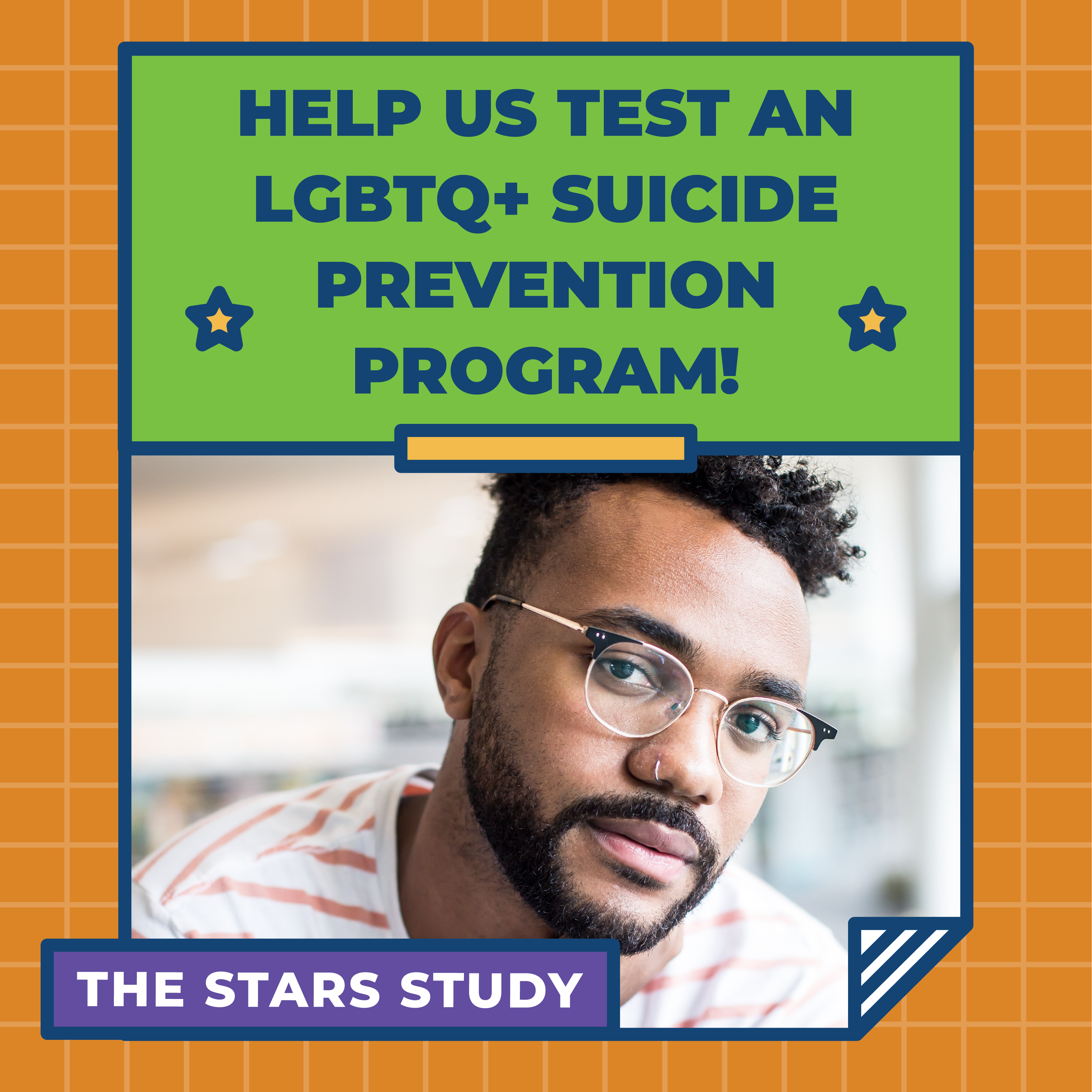

Supplement: Supplementary file 2 — Supplementary file2 (PNG 6741 KB) [file 10508_2026_3438_MOESM2_ESM.png]

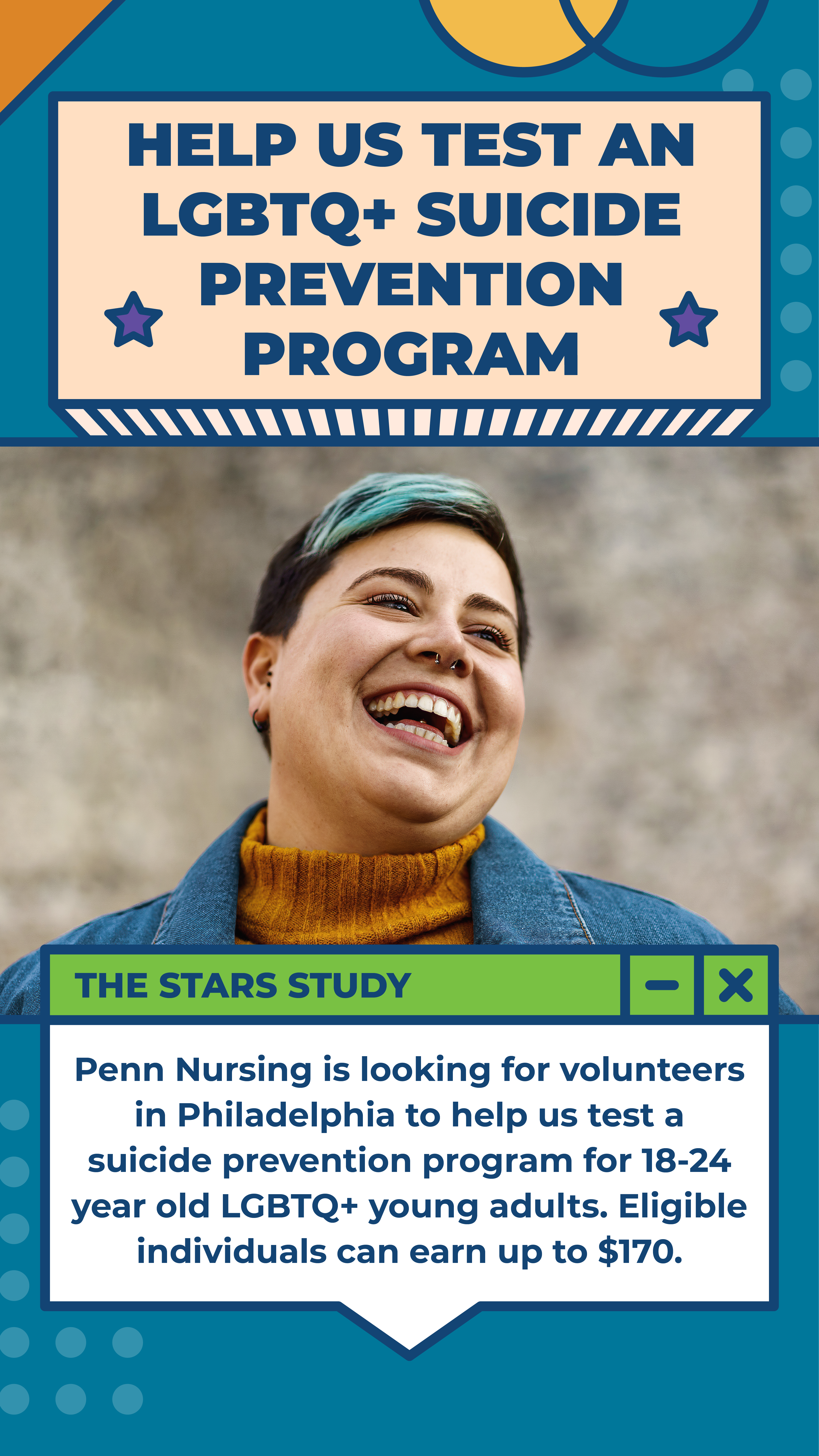

Supplement: Supplementary file 3 — Supplementary file3 (PNG 16491 KB) [file 10508_2026_3438_MOESM3_ESM.png]
